# Culture positivity may correlate with long-term mortality in critically ill patients

**DOI:** 10.1186/s12879-021-06898-8

**Published:** 2021-11-26

**Authors:** Wei-Fan Ou, Li-Ting Wong, Chieh-Liang Wu, Wen-Cheng Chao

**Affiliations:** 1 Division of Chest Medicine, Department of Internal Medicine, Taichung Veterans General Hospital, Taichung, Taiwan; 2 Department of Medical Research, Taichung Veterans General Hospital, Taichung, Taiwan; 3 Department of Critical Care Medicine, Taichung Veterans General Hospital, No, 1650, Section 4, Taiwan Boulevard, Xitun District, Taichung, 40705 Taiwan; 4 Department of Computer Science, Tunghai University, Taichung, Taiwan; 5 Department of Automatic Control Engineering, Feng Chia University, Taichung, Taiwan; 6 Department of Industrial Engineering and Enterprise Information, Tunghai University, Taichung, Taiwan; 7 Artificial Intelligence Studio, Taichung Veterans General Hospital, Taichung, Taiwan; 8 Big Data Center, Chung Hsing University, Taichung, Taiwan

**Keywords:** Critical illness, Long-term outcome, Survival analysis, Culture positivity

## Abstract

**Background:**

The long-term outcome is currently a crucial issue in critical care, and we aim to address the association between culture positivity and long-term mortality in critically ill patients.

**Methods:**

We used the 2015–2019 critical care database at Taichung Veterans General Hospital and Taiwanese nationwide death registration files. Multivariable Cox proportional hazards regression model was conducted to determine hazard ratio (HR) and 95% confidence interval (CI).

**Results:**

We enrolled 4488 critically ill patients, and the overall mortality was 55.2%. The follow-up duration among survivors was 2.2 ± 1.3 years. We found that 52.6% (2362/4488) of critically ill patients had at least one positive culture during the admission, and the number of patients with positive culture in the blood, respiratory tract and urinary tract were 593, 1831 and 831, respectively. We identified that a positive culture from blood (aHR 1.233; 95% CI 1.104–1.378), respiratory tract (aHR 1.217; 95% CI 1.109–1.364) and urinary tract (aHR 1.230; 95% CI 1.109–1.364) correlated with an increased risk of long-term mortality after adjusting relevant covariates.

**Conclusions:**

Through linking two databases, we found that positive culture in the blood, respiratory tract and urinary tract during admission correlated with increased long-term overall mortality in critically ill patients.

**Supplementary Information:**

The online version contains supplementary material available at 10.1186/s12879-021-06898-8.

## Background

The long-term outcome is currently an emerging research niche in critical care medicine due to increasing awareness of sequelae after the critical illness [1, 2]. However, early determinants for long-term outcome in critically ill patients remains largely unexplored. Growing studies, particularly microbiome-associated studies, have shown the prolonged microbial-associated impact in critically ill patients [3–5]. A number of studies have investigated the association between culture positivity and mortality in critically ill patients; however, discordant evidence were found, and the discrepancy mainly result from the distinct follow-up duration among these studies [6–9]. Li et al., analysing seven studies involving 22,655 patients, recently reported that culture positivity was not associated with short-term mortality; however, the hospital-day and ventilator-day were longer in culture-positive patients than those in culture-negative patients, implicating a potential long-term impact of the microbial factor in critically ill patients [10]. We hence aimed to explore the association between culture positivity during admission and long-term mortality in critically ill patients. In the present study, we linked the critical care database at Taichung Veteran General Hospital (TCVGH) with the death registration data of the Taiwanese National Health Insurance Research Database (NHIRD) to address the association of microbial factors, including pathogen and culture sites, and long-term mortality in critically ill patients.

## Methods

### Ethical approval

The study was performed in accordance with the Declaration of Helsinki. This study was approved by the Institutional Review Board of the Taichung Veterans General Hospital (TCVGH: SE20249B#1), and informed consent was waived due to the data were deidentified prior to analyses.

### Study population

We conducted this retrospective cohort study by including data of consecutive patients who were admitted to medical ICUs at TCVGH, a referral hospital with 1,500 beds and three medical ICUs in central Taiwan, between 2015-Jan and 2019-June. In patients who had been admitted to the ICU for more than one time, the first ICU admission was used as the index ICU admission.

### Data collection and definition of variables

We used two data sources: the critical care data warehouse at TCVGH and the death registry profile in Taiwan. Data with respects of demographic data, Charlson comorbidity index (CCI), etiologies for ICU admission, discharge diagnoses, Acute Physiology and Chronic Health Evaluation (APACHE) II score, managements including renal replacement therapy as well mechanical ventilation during admission and microbial data were obtained from the TCVGH critical care data warehouse [11, 12]. The presence of shock was defined by the requirement of vasopressor, and the immunocompromised patient was defined by one of the following conditions, including active haematological disease or solid tumour under therapy and receiving immunosuppressants due to autoimmune disease or organ transplant recipient [13]. The main outcome of interest in this study was the overall mortality following ICU admission. The date-of-death of enrolled critically ill patients was retrieved from the death registration profile of the NHIRD in Taiwan [14]. Given that the Taiwanese National Health Insurance (NHI) is a single-payer and compulsory nationwide insurance program with 99.9% coverage of the Taiwanese population in 2019, the date-of-death in the present study should be precise.

### Microbiological cultures

The exposure of interest in this study was the positive culture of samples obtained during the index admission. The sites of infection were grouped by blood, respiratory tract (i.e., tracheal aspirate, bronchoalveolar lavage fluid, and pleural effusion), abdomen (i.e., bile, ascites, and peritoneal drainage), skin and soft tissue (i.e., wound and discharge) and urinary tract (i.e., midstream urine, urine via urinary catheter, and urine through cystostomy/percutaneous nephrostomy) during the index ICU admission [15]. The microorganisms were categorised by Gram-positive cocci (GPC), Gram-negative bacilli (GNB), or Fungi including Candida and *Aspergillus* [15]. Coagulase-negative Staphylococcus (CoNS) was not included for analysis given that CoNS tend to be contaminants. We also identified patients who had the positive culture with multiple drug resistant organisms (MDRO), including methicillin-resistant *Staphylococcus aureus*, vancomycin-resistant *enterococci* and carbapenem-resistant Gram-negative bacilli [15, 16].

### Statistical analyses

Descriptive results were presented as means ± standard deviation or number (percentages).

Kaplan–Meier analysis was used for the association between microbial culture results and mortality. The Cox proportional hazards model was used to estimate hazard ratios (HRs) and 95% confidence intervals (CIs) for mortality after adjustment for age, sex, CCI, and other potential cofounders including early fluid balance as we have shown as a predictor for long-term mortality in our previous study [17]. Statistical analyses were two-sided, and the level of significance was set with 0.05. Data analysis were conducted using R version 3.6.0.

### Sensitivity and subgroup analyses

We further used distinct numbers of pathogens to define culture positivity and to test the robustness of the association between culture positivity and long-term mortality in critically ill patients. Additionally, we used the Wald test to check the modification effect by covariates, including age, sex, and the presence of immunocompromised conditions.

## Results

### Characteristics of the enrolled subjects with critical illness

**Fig. 1:**
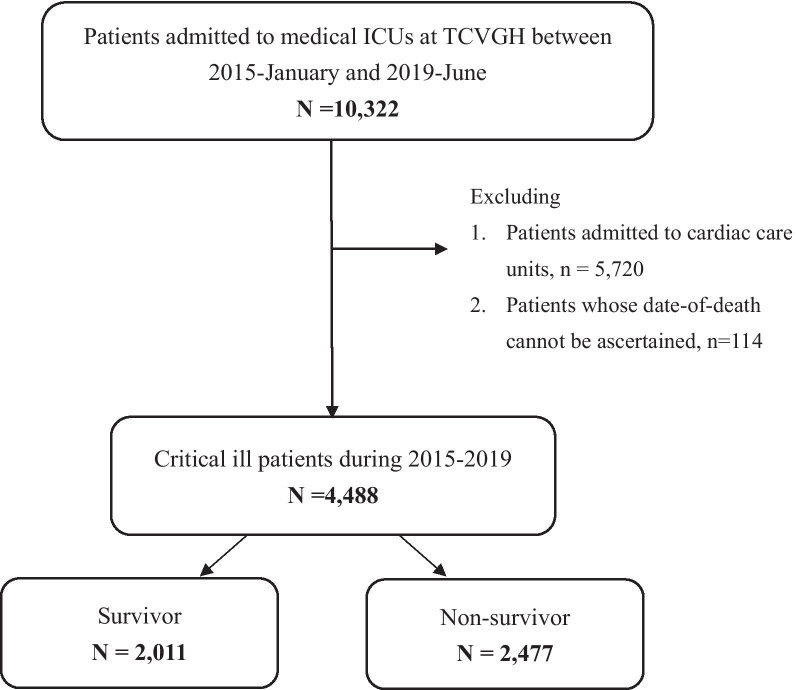
Flowchart of subject enrollment. *TCVGH* Taichung Veterans General Hospital, *ICU* intensive care unit

A total of 4488 critically ill patients were eligible for analyses; the mean age was 66.4 ± 16.4 years, with 63.9% of them were male (Table 1 and Fig. 1). The overall mortality was 55.2% (2,477/4,488), and the follow-up duration among survivors was 2.2 ± 1.3 years. In detail, the in-hospital mortality rate, 90-day and 1-year mortality was 28.1%, 38.0%, and 47.2%, respectively. The post-discharge 1-year mortality rate among critically ill patients who survived after the ICU admission was 26.5% (857/3,228). Compared with survivors, non-survivors were older (69.7 ± 15.5 vs. 62.3 ± 16.6 years, p < 0.01), were more likely to be male (66.0% vs. 61.3%, p < 0.01), had higher CCI (2.7 ± 1.6 vs. 2.0 ± 1.5, p < 0.01), and a lower body mass index (BMI) (23.9 ± 4.7 vs. 24.9 ± 4.7, p < 0.01). Non-survivors were more likely have the immunocompromised condition (23.3% vs. 18.7%, p < 0.01) and were admitted to ICU due to sepsis with acute respiratory failure (67.3% vs. 45.5%) than those in survivors. Moreover, non-survivors had a higher APACHE II score (27.7 ± 7.0 vs. 21.9 ± 6.7, p < 0.01) and were more likely to have a shock (59.6% vs. 29.4%, p < 0.01), to receive mechanical ventilation for more than 3 days (82.5% vs. 62.2%, p < 0.01) and to receive renal replacement therapy (21.5% vs. 8.7%, p < 0.01) (Table 1). Taken together, these data demonstrated high long-term mortality among critically ill patients with high disease severity and indicated the essential need to address the early determinants for long-term mortality in critically ill patients.

**Table 1:** Characteristics of the 4,488 enrolled critically ill patients divided by overall mortality

	All	Non-survivors	Survivors	*p* value
	(N = 4488)	(N = 2477)	(N = 2011)	
Basic characteristics				
Age, years	66.4 ± 16.4	69.7 ± 15.5	62.3 ± 16.6	< 0.01
Sex (male)	2866 (63.9%)	1634 (66.0%)	1232(61.3%)	< 0.01
Body mass index	24.4 ± 4.7	23.9 ± 4.7	24.9 ± 4.7	< 0.01
Charlson comorbidity index	2.4 ± 1.6	2.7 ± 1.6	2.0 ± 1.5	< 0.01
Immunocompromised patients	953 (21.2%)	578 (23.3%)	375(18.7%)	< 0.01
Malignancy, active	601 (13.4%)	508 (20.5%)	93 (4.6%)	< 0.01
Solid tumor, active	427 (9.5%)	372 (15.0%)	55 (2.7%)	< 0.01
Hematological malignancy	174 (3.9%)	136 (5.5%)	38 (1.9%)	< 0.01
Autoimmune diseases	107 (2.4%)	54 (2.2%)	53 (2.6%)	0.32
Organ transplant recipients	15 (0.3%)	7 (0.3%)	8 (0.4%)	0.51
Follow-up duration, years	1.2 ± 1.3	0.4 ± 0.8	2.2 ± 1.3	< 0.01
Etiology for ICU admission				
Sepsis with acute respiratory failure	2584 (57.6%)	1668 (67.3%)	916 (45.5%)	< 0.01
Acute neurological conditions	431 (10.6%)	170 (7.0%)	261 (13.0%)	
Acute cardiac conditions	190 (4.2%)	42 (1.7%)	148 (7.4%)	
Acute gastrointestinal condition	174 (3.9%)	109 (4.4%)	65 (3.2%)	
Acute renal conditions	121 (2.7%)	56 (2.3%)	65 (3.2%)	
Major surgery	101 (2.3%)	35 (1.4%)	66 (3.3%)	
Cardiac arrest	25 (0.6%)	22 (0.9%)	3 (0.2%)	
Others	862 (19.2%)	375 (15.1%)	487 (24.2%)	
Severity and managements				
APACHE II score	25.1 ± 7.5	27.7 ± 7.0	21.9 ± 6.7	< 0.01
Presence of shock	2068 (46.1%)	1476 (59.6%)	592 (29.4%)	< 0.01
Receiving mechanical ventilation	3295 (73.4%)	2044 (82.5%)	1,251 (62.2%)	< 0.01
Renal replacement therapy (RRT)				
Temporal RRT during admission	708 (15.8%)	533 (21.5%)	175 (8.7%)	< 0.01
RRT for ESRD	125 (2.8%)	68 (2.8%)	57 (2.8%)	0.86
Fluid balance, day 1–3, mL	717.6 ± 3727.4	1408.7 ± 4106.1	− 133.6 ± 2988.9	< 0.01
Microbiologic data				
Positive culture, any culture site	2362 (52.6%)	1608 (64.9%)	754 (37.5%)	< 0.01
Blood	631 (14.1%)	491 (19.8%)	140 (7.0%)	< 0.01
Respiratory Tract	1823 (40.6%)	1267(51.2%)	556 (27.7%)	< 0.01
Urinary Tract	831 (18.5%)	607 (24.5%)	224 (11.1%)	< 0.01
Other sites	160 (3.6%)	111 (4.5%)	49 (2.4%)	< 0.01
Positive MDRO^1^	1286 (28.7%)	925 (37.3%)	361 (18.0%)	< 0.01
Outcomes				
ICU-stay, days	9.9 ± 8.4	11.3 ± 8.8	8.3 ± 7.6	< 0.01
Hospital-stay, days	24.2 ± 19.1	26.4 ± 19.7	21.6 ± 17.8	< 0.01
Ventilator-day	9.8 ± 9.1	10.8 ± 9.6	8.1 ± 8.0	< 0.01
Mortality at distinct time points				
In-hospital mortality	1260 (28.1%)	1260 (50.9%)	NA	NA
90-day mortality	1707 (38.0%)	1707 (68.9%)	NA	NA
1-year mortality	2117 (47.2%)	2117 (85.5%)	NA	NA

^1^MDRO, included methicillin-resistant *Staphylococcus aureus*, vancomycin-resistant *Enterococci*, and carbapenem-resistant Gram-negative bacilli. *APACHE II* acute physiology and chronic health evaluation, *RRT* renal replacement therapy, *ESRD* end-stage renal disease, *MDRO* multidrug-resistant organism, *ICU* intensive care unit, *NA* not applicable

### Main pathogens in distinct culture sites among critically ill patients

We found that 52.6% (2362/4488) of enrolled critically ill patients had at least one positive culture, and the number of subjects with positive culture in the blood, respiratory tract, and urinary tract were 593, 1831, and 831, respectively (Table 2). Non-survivors tended to have a higher proportion of MDRO than those in survivors (37.3% vs. 18.0%, p < 0.01). The leading Gram-positive cocci was *Staphylococcus aureus* (n = 292, 50.0%), followed by *Enterococcus faecium* (n = 131, 22.4%) and *Enterococcus faecalis* (n = 55, 9.4%). Among the Gram-negative bacilli, the 5 leading pathogens were *Pseudomonas aeruginos*a (n = 513, 29.4%), *Klebsiella pneumoniae* (n = 562, 32.7%), *Acinetobacter baumannii* (n = 402, 19.9%), *Escherichia coli* (n = 342, 19.9%) as well as *Enterobacter cloacae* (n = 85, 4.9%), and the aforementioned data were in line with the nationwide surveillance of pathogens in healthcare facilities of Taiwan [18]. Fungal infection currently is an emerging issue in critically ill patients worldwide [19], and we found that the number of patients had positive culture for *Candida*/Yeast and *Aspergillus* were 14.1% (632/4488) and 2.4% (108/4488), respectively. 1.4% (64/4488) of critically ill patients had Candidemia, incluidng *Candida albican* (n = 36) followed by *Candida glabrata* (n = 15) and *Candida tropicalis* (n = 11). Additionally, 108 critically ill patients have a positive culture for *Aspergillus*, mainly in the respiratory tract.

**Table 2:** Pathogens identified in the cultures of 2,362 patients during their index admission

	Total		Blood		Respiratory tract		Urinary tract		Others	
	(N = 2362)		(N = 593)		(N = 1831)		(N = 831)		(N = 352)	
	n	%	n	%	n	%	n	%	n	%
Gram-positive cocci	N = 584		N = 211		N = 246		N = 143		N = 89	
* Staphylococcus aureus*	292	50.0%	92	43.6%	219	89.0%	6	4.2%	30	33.7%
* Enterococcus faecium*	131	22.4%	39	18.5%	2	0.8%	78	54.5%	26	29.2%
* Enterococcus faecalis*	55	9.4%	8	3.8%	0	0.0%	38	26.6%	13	14.6%
Gram-negative bacilli	N = 1720		N = 389		N = 1379		N = 383		N = 215	
* Pseudomonas aeruginosa*	513	29.8%	42	10.8%	446	32.3%	65	17.0%	33	15.3%
* Klebsiella pneumoniae*	562	32.7%	126	32.4%	415	30.1%	95	24.8%	51	23.7%
* Acinetobacter baumannii*	402	23.4%	41	10.5%	357	25.9%	33	8.6%	27	12.6%
* Escherichia coli*	342	19.9%	96	24.7%	104	7.5%	151	39.4%	51	23.7%
* Enterobacter cloacae*	85	4.9%	19	4.9%	53	3.8%	9	2.3%	16	7.4%
* Serratia marcescens*	52	3.0%	9	2.3%	41	3.0%	2	0.5%	3	1.4%
* Proteus mirabilis*	40	2.3%	8	2.1%	16	1.2%	12	3.1%	11	5.1%
* Enterobacter aerogenes*	34	2.0%	4	1.0%	24	1.7%	7	1.8%	6	2.8%
* Haemophilus influenzae*	31	1.8%	0	0.0%	31	2.2%	0	0.0%	0	0.0%
*Candida*/Yeast	N = 632		N = 64		N = 264		N = 367		N = 38	
* Candida albicans*	436	69.0%	36	5.7%	200	31.6%	231	36.6%	31	4.9%
* Candida glabrata*	126	19.9%	15	2.4%	27	4.3%	81	12.8%	10	1.6%
* Candida tropicalis*	77	12.2%	11	1.7%	35	5.5%	35	5.5%	6	0.9%
* Candida parapsilosis*	12	1.9%	0	0.0%	7	1.1%	5	0.8%	0	0.0%
* Candida krusei*	2	0.3%	0	0.0%	2	0.3%	0	0%	0	0.0%
Yeast	45	7.1%	45	7.1%	1	0.2%	44	7%	0	0.0%
*Aspergillus*	N = 108		N = 0		N = 107		N = 0		N = 1	
* Aspergillus fumigatus*	58	53.7%	0	0.0%	58	54.2%	0	0.0%	0	0.0%
* Aspergillus flavus*	31	28.7%	0	0.0%	30	28.0%	0	0.0%	1	100%
* Aspergillus niger*	10	9.3%	0	0.0%	10	9.3%	0	0.0%	0	0.0%
* Aspergillus terreus*	9	8.3%	0	0.0%	9	8.4%	0	0.0%	0	0.0%

### Association between microbial culture and long-term mortality

**Fig. 2:**
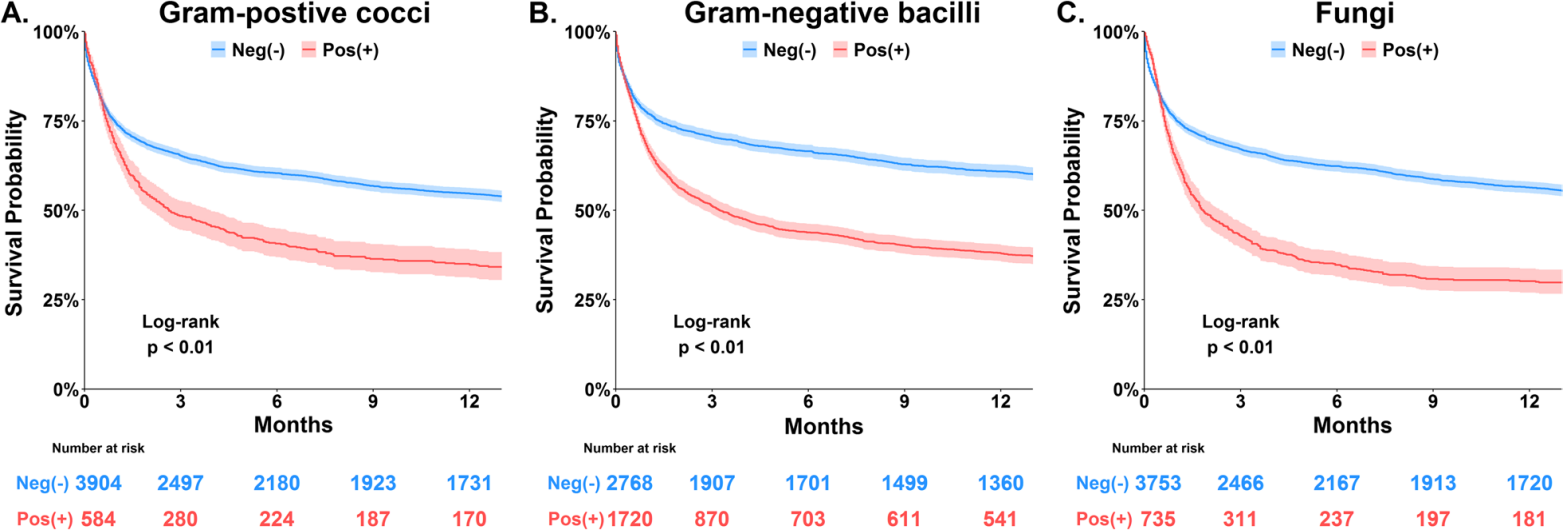
Kaplan–Meier survival curves for patients with and without the pathogens. **A** Gram-positive cocci, **B** Gram-negative bacilli, and **C** Fungi including *Candida*/yeast and *Aspergillus*

**Fig. 3:**
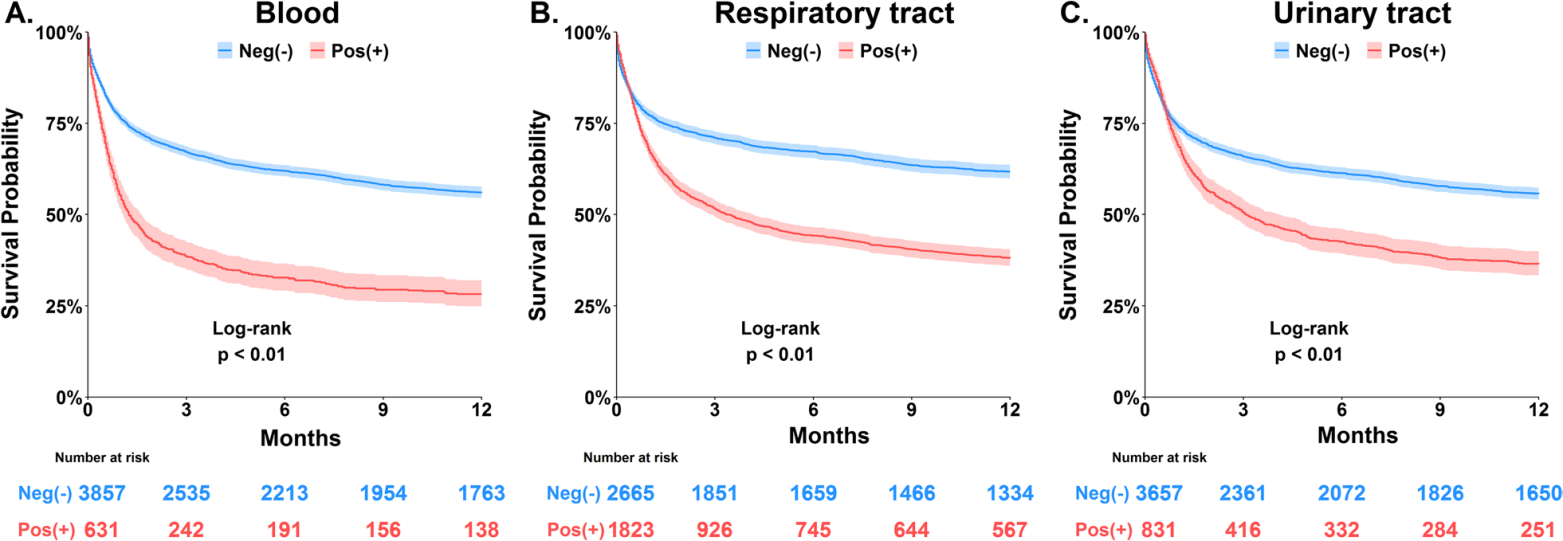
Kaplan–Meier survival curves for patients categorised by culture sites. **A** Blood, **B** Respiratory tract, and **C** Urinary tract

We used Kaplan–Meier analyses to examine the correlation between distinct pathogens as well as culture positivity in distinct culture sites and long-term mortality (Figs. 2, 3). Notably, we found that the mortality impact of culture positivity in blood, respiratory tract and urinary tract appeared to be lasting for approximately 3–6 months (Fig. 3). We then used the multivariable Cox proportional hazards model to determine the independent mortality association of culture positivity in distinct sites.

We identified that a positive culture from blood (aHR 1.233; 95% CI 1.104–1.378), respiratory tract (aHR 1.217; 95% CI 1.109–1.337) and urinary tract (aHR 1.230; 95% CI 1.109–1.364) correlated with an increased risk of long-term mortality after adjusting for age (aHR 1.008; 95% CI 1.005–1.010 per 1-year increment), male gender (aHR 1.201; 95% CI 1.104–1.308), BMI (aHR, 1.035; 95% CI 1.026–1.045), CCI (aHR 1.120; 95% CI 1.093–1.148), APACHE II score (aHR, 1.053; 95% CI 1.046–1.061 per 1-point increment), and early fluid balance (aHR 1.050; 95% CI 1.039–1.061 per 1 L increment) (Table 3). We noted that receiving renal replacement therapy (aHR 1.469; 95% CI 1.324–1.631), presence of shock (aHR 1.521; 95% CI 1.387–1.667) and immunocompromised condition (aHR 2.341; 95% CI 2.123–2.581) appeared to be relatively strong predictors for long-term mortality; therefore, we further checked the interaction effect of these variables. We found that the association between culture positivity and long-term mortality were higher in those without shock, immunocompromised condition and renal replacement therapy than comparable groups. (Additional file 1: Table S1). In sensitivity analysis, we found that culture positivity with distinct numbers of pathogens and cites was associated with high long-term mortality at a dose–response manner (Table 4).

**Table 3:** Cox proportional hazards regression for long-term mortality

Characteristics	Univariable Univariable		Multivariable	
	HR (95% CI)	*p* value	HR (95% CI)	*p* value
Age, per 1 year increment	1.017 (1.014–1.019)	< 0.001	1.008 (1.005–1.010)	< 0.001
Male gender	1.136 (1.046–1.235)	0.003	1.201 (1.104–1.308)	< 0.001
Body mass index, per 1 decrement	1.025 (1.016–1.034)	< 0.001	1.035 (1.026–1.045)	< 0.001
Charlson comorbidity index, per 1 increment	1.192 (1.166–1.219)	< 0.001	1.120 (1.093–1.148)	< 0.001
APACHE II, per 1 increment	1.095 (1.089–1.102)	< 0.001	1.053 (1.046–1.061)	< 0.001
Receiving mechanical ventilation	2.093 (1.887–2.322)	< 0.001	1.068 (0.953–1.198)	0.256
Fluid overload, day 1–3, per 1 L increment	1.096 (1.085–1.106)	< 0.001	1.050 (1.039–1.061)	< 0.001
Receiving renal replacement therapy	2.309 (2.097–2.543)	< 0.001	1.469 (1.324–1.631)	< 0.001
Presence of shock	2.565 (2.366–2.780)	< 0.001	1.521 (1.387–1.667)	< 0.001
Immunocompromised patients	2.418 (2.200–2.658)	< 0.001	2.341 (2.123–2.581)	< 0.001
Positive culture of MDRO^1^	1.774 (1.635–1.925)	< 0.001	1.008 (1.005–1.010`)	< 0.001
Culture site				
Blood	2.141 (1.938–2.364)	< 0.001	1.233 (1.104–1.378)	< 0.001
Respiratory tract	1.873 (1.731–2.028)	< 0.001	1.217 (1.109–1.337)	< 0.001
Urinary tract	1.648 (1.504–1.807)	< 0.001	1.230 (1.109–1.364)	< 0.001
Skin and soft tissue	1.519 (1.198–1.927)	0.001	0.943 (0.741–1.200)	0.633
Abdomen	1.835 (1.516–2.223)	< 0.001	1.028 (0.846–1.249)	0.780

^1^MDRO, included methicillin-resistant *Staphylococcus aureus*, vancomycin-resistant *Enterococci*, and carbapenem-resistant Gram-negative bacilli. *HR* hazard ratio, *CI* confidence interval, *APACHE* acute physiology and chronic health evaluation, *MDRO* multidrug-resistant organism

**Table 4:** Sensitivity analysis using distinct definitions of culture positivity to estimate the mortality risk

Distinct definitions of culture positivity	Adjusted HR* (95% CI)
Pathogens	
At least one pathogen (main finding)	1.27 (1.15–1.42)
At least two pathogens	1.40 (1.24–1.58)
At least three pathogens	1.52 (1.35–1.71)
Culture sites	
At least one site (main finding)	1.29 (1.17–1.42)
At least two sites	1.44 (1.28–1.62)
At least three sites	1.67 (1.44–1.94)

*Adjusted covariates including variables listed in Table 3. *CI* confidence interval

## Discussion

The long-term outcome is an emerging research niche in critical care medicine, and identifying early determinants for long-term mortality is an unmet need. In the present study, we linked two databases to address the association between culture positivity and long-term mortality in critically ill patients. We found that culture positivity in the blood, respiratory tract and urinary tract were independently associated with long-term mortality after the adjustment of a number of covariates. We also identified long-term mortality relevant predictors, and these findings should be crucial for risk stratification that may in turn to ensure implementation of preventive strategies in patients who survived from critical illness.

Long-term outcome in critically ill patients is currently an emerging research niche due to increased awareness of sequelae among patients who survived from critical illness [1, 20]. Moitra et al. reported that the 1-year mortality among 34,696 ICU survivors in Medicare claim was 26.6% [21], and the finding was similar to our data that 1-year mortality among ICU survivors was approximately 26.5% (857/3228). In addition to recognising the crucial role of long-term outcomes in critically ill patients, a number of studies, including our previous study, have identified the early determinant for the long-term outcome [17, 22]. Our recently published studies also revealed that an early positive fluid balance status was associated with high long-term mortality in critically ill cancer patients [17]. In the present study, we further discovered that culture positivity was associated with long-term mortality after adjustment of covariates including early fluid status in critically ill patients. These early predictors for long-term mortality provide clinical evidence for risk stratification that should ensue early implementation of nutritional, physical, and psychological support after critical illness [23].

The association between culture positivity and outcomes, particularly long-term outcomes, in critically ill patients remains inconclusive. A number of studies have explored the short-term impact of culture positivity in critically ill patients [6, 7]. Phua et al*.* conducted a single-centre study in Singapore to compare the outcome between 415 culture-negative and 586 culture-positive patients with severe sepsis during 2004–2009 [6]. They found lower hospital mortality in the culture-negative group than those in the culture-positive group (35.9% vs. 44.0%, p = 0.01) in the univariable analysis, but culture positivity was no longer associated with mortality after adjusting covariates [6]. Kim et al. recently used the 2014–2018 septic shock registry at a Korean tertiary referral centre to explore the association between culture positivity and 90-day mortality among 1718 patients with septic shock, mainly result from biliary tract infection and pneumonia and found similar 90-day mortality in culture-positive and culture-negative patients (32.2% vs. 32.7%, p = 0.83) [7]. Few studies have explored the association between culture positivity and long-term outcome in critically ill patients.

In line with our finding, Francisco et al., conducting a single hospital study in Portugal involving 1013 patients admitted with severe infection during 2008–2009, reported the 5-year mortality was 37%, and culture-positive was independent predictors (aOR 1.48; 95% CI 1.07–2.06) for 5-year mortality [24]. Similarly, Nannan Panday et al., investigating 2659 septic patients admitted to 34 hospitals in the Netherlands during 2014–2016, found that 42.6% of them was culture-positive and culture positivity was associated with high 90-day mortality (relative risk 1.41, 95% CI 1.15–1.71) [9]. To address the distinct association of culture positivity with the short-term and long-term outcome, we conducted further analysis of predictors for 30-day mortality of the enrolled 4,610 critically ill patients in the present study (Additional file 1: Table S2). Compared with data in the present study addressing long-term mortality (Table 3), the high comorbidity, disease severity and management had a similar association with 30-day mortality, whereas culture positivity was unrelated with the 30-day mortality. Therefore, culture positivity tends to be associated with long-term outcome, instead of short-term outcome. These evidence highlight the previously less recognised impact on long-term mortality of culture positivity in critically ill patients.

The damaged microbiome with low diversity may be a plausible biological basis of the prolonged impact of culture positivity in critically ill patients [3, 25]. The altered microbiome has being increasingly recognised not only to be merely a disrupted mucosal barrier but also to affect immune function through regulating the expansion of pathogenic bacteria and modulating the immune response in critically ill patients [3, 26]. The altered microbiome in critically ill patients may be attributed to not only the infectious disease as well as antibiotics but also critical ill relevant medications, including proton pump inhibitors, vasopressors, opioids, and analgesics [27]. Notably, the impact of disruption of the gut microbiome has been found to be long-lasting, and it may take longer than 6 months for recovery [5, 28]. Furthermore, accumulating evidence have shown the cross-talk between the microbiome and immune response, particularly CD4 T cell [29, 30]. Recently, Fay et al*.* demonstrated that the gut microbiome may affect the immunophenotype and survival from sepsis in the mouse sepsis model with cecal ligation and puncture (CLP) [29]. In brief, Fay et al. identified distinct microbiome between genetically identical age- and gender-matched mice from Jackson Laboratory (Jax) and Charles River Laboratory (CR) and distinct 7-day mortality after CLP (90% in Jax vs. 53% in CR). In septic CR mice, an altered immunophenotype with high IFN − γ + CD4 + T cells and effector/central memory CD4 + T cells in the spleen, Peyer’s patches, and mesenteric lymph nodes were found. After the cohouse for 3 weeks, the differences in immunophenotype and microbiome no longer existed, and the post-CLP mortality of cohoused Jax mice improved, implicating the microbiome tends to affect immunophenotype and mortality in sepsis [29]. Xu et al*.* conducted a prospective cohort study with 98 critically ill neurological patients and 84 matched healthy subjects found that increased intestinal *Enterobacteriales* and *Enterobacteriaceae* within the first week were associated with high 180-day mortality [31]. The aforementioned animal and clinical evidence highlight that early alternation of the gut microbiome has a prolonged immunological impact and may affect the long-term outcome in critically ill patients.

In the present study, we found similar trends among the three major types of pathogen pathogens (Fig. 2). The mortality association was also similar in distinct culture sites (Fig. 3), although the strength of association in skin soft tissue and abdomen did not reach statistical significance due to a limited number of patients (Table 3 and Additional file 1: Fig. S1). Herein, the proportion of a positive MDRO was higher in non-survivors than those in survivors. However, the presence of MDRO was no longer significantly associated with long-term mortality after adjusting for disease severities and the other covariates, and more studies are warranted to further explore the long-term impact of drug-resistant organisms among critically ill patients in the future. Intriguingly, the mortality impact of culture positivity in critically ill patients mainly existed within approximately 6 months of ICU admission, and the finding appears in line with studies have discovered that the altered microbiome in patients with critical illness tended to be restored approximately 6 months after critical illness [32, 33].

In this study, we found that 1.4% (64/4488) of critically ill patients had Candidemia, and invasive fungal infection is currently an increasing threat among critically ill patients in Taiwan and the world [34]. The aforementioned relatively high prevalence of invasive fungal infection in this study may at least partly result from the high proportion of immunocompromised patients [35]. Given that immunocompromised status appeared to be a crucial predictor for long-term mortality in the present study, we hence further explored the modification effect of immunocompromised status on the association between culture positivity and long-term mortality in critically ill patients. Intriguingly, we found that the association between culture positivity and mortality was stronger in patients without immunodeficiency than that in immunocompromised patients (Additional file 1: Table S1).

There are limitations that merit discussion. First, data from this single-centre study may not be generalisable to other healthcare settings. However, the data analysed are real-world data obtained in routine critical care, and the issue of generalisation should be at least partly mitigated. Second, the causal inference could not be drawn given the observation nature of this study. Third, sampling bias might be a concern given that decisions for the microbiological test were made by individual intensivists; however, the administration of intensivists in the study hospital should mitigate the aforementioned concern. Fourth, we could not delineate true, colonised, or contaminated pathogens in this claims-based research.

## Conclusions

In conclusion, the identification of early determinants for long-term mortality is a research niche in critical care medicine. Currently, there are discrepant evidences regarding the association between culture positivity and outcome, particularly long-term outcome, in critically ill patients. We linked two databases and found that culture positivity in the blood, respiratory tract and urinary tract were associated with long-term mortality in critically ill patients. Our findings highlight the previous under-recognised role of culture positivity in critically ill patients, and more studies are warranted to clarify the biological mechanisms.

## Supplementary Information

**Additional file 1: Table S1**. Effect modification of variables on the association between culture positivity and risk of mortality.** Table S2**. Cox proportional hazards regression for 30-day mortality.** Figure S1**. Kaplan-Meier survival curves for patients categorised by culture sites.

## Data Availability

The data underlying this article will be shared on request to the corresponding author.
