# Supplementary material for: Culture positivity may correlate with long-term mortality in critically ill patients
Source: BMC Infect Dis. 2021 Nov 26;21:1188. doi: 10.1186/s12879-021-06898-8 (PMC8620521; doi:10.1186/s12879-021-06898-8)
Supplement: Supplementary file 1 — Additional file 1: Table S1. Effect modification of variables on the association between culture positivity and risk of mortality. Table S2. Cox proportional hazards regression for 30-day mortality. Figure S1. Kaplan-Meier survival curves for patients categorised by culture sites. [file 12879_2021_6898_MOESM1_ESM.pdf]

Supplemental Table 1. Effect modification of variables on the association between culture positivity and risk of mortality

**Groups**	**adjHR (95% CI)**	***p* value**	***p* for interaction**
**Age, years**			0.322
≤ 50	2.073 (1.645-2.613)	<0.001	
> 50	2.037 (1.864-2.227)	<0.001	
**Sex**			0.676
Female	2.021 (1.751-2.333)	<0.001	
Male	2.133 (1.927-2.361)	<0.001	
**Presence of shock**			<0.001
No	2.440 (2.151-2.767)	<0.001	
Yes	1.374 (1.135-1.663)	0.001	
Renal replacement therapy			0.001
No	2.101 (1.915-2.304)	<0.001	
Yes	1.580 (1.335-1.870)	<0.001	
**Immunodeficiency**			<0.001
No	2.308 (2.097-2.540)	<0.001	
Yes	1.242 (1.111-1.388)	<0.001	
**Immunodeficiency type**			0.652
No	2.308 (2.097-2.540)	<0.001	
Solid tumor, active	1.172 (0.955-1.438)	0.129	
Hematological malignancy	2.016 (1.403-2.896)	<0.001	
Autoimmune diseases	3.224 (1.775-5.855)	<0.001	
Organ transplant recipients	4.278 (0.938-19.52)	0.061	

Abbreviations: HR, hazard ratio.

Supplemental Table 2. Cox proportional hazards regression for 30-day mortality.

Characteristics	Univariable		Multivariable	
	**HR (95% C.I.)**	***p* value**	**HR (95% C.I.)**	***p* value**
Age, per 1 year increment	1.002 (0.998-1.005)	0.355	0.995 (0.991-0.999)	0.007
Male gender	1.075 (0.954-1.211)	0.236	1.120 (0.993-1.265)	0.065
Body mass index, per 1 decrement	0.993 (0.981-1.004)	0.200	1.017 (1.004-1.030)	0.010
Charlson comorbidity index, per 1 increment	1.127 (1.090-1.164)	<0.001	1.053 (1.015-1.092)	0.006
APACHE II, per 1 increment	1.117 (1.107-1.126)	<0.001	1.077 (1.066-1.088)	<0.001
Receiving mechanical ventilation	1.878 (1.615-2.184)	<0.001	0.821 (0.696-0.968)	0.019
Fluid overload, day 1-3, per 1L increment	1.118 (1.106-1.130)	<0.001	1.059 (1.045-1.072)	<0.001
Receiving renal replacement therapy	2.840 (2.510-3.214)	<0.001	1.558 (1.361-1.784)	<0.001
Presence of shock	4.060 (3.571-4.616)	<0.001	2.261 (1.956-2.615)	<0.001
Immunocompromised patients	2.854 (2.525-3.227)	<0.001	2.239 (1.972-2.542)	<0.001
Culture site				
Blood	2.118 (1.854-2.420)	<0.001	1.126 (0.975-1.301)	0.107
Respiratory tract	1.437 (1.283-1.609)	<0.001	0.957 (0.847-1.080)	0.477
Urinary tract	1.190 (1.036-1.366)	0.014	0.962 (0.831-1.113)	0.599
Skin and soft tissue	0.850 (0.558-1.296)	0.451	0.544 (0.355-0.833)	0.005
Abdomen	1.423 (1.077-1.878)	0.013	0.774 (0.583-1.027)	0.076

Abbreviations: HR, hazard ratio; C.I., confidence interval; APACHE, acute physiology and chronic health evaluation.

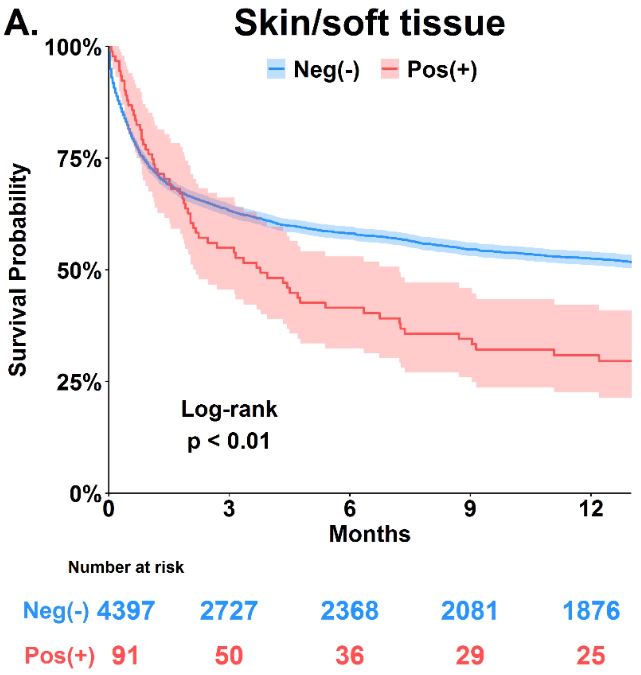

Supplemental Figure 1. Kaplan-Meier survival curves for patients categorised by culture sites. (A) skin and soft tissue and (B) abdomen
